# The mental health of brazilian university hospital’s workers in times of COVID-19

**DOI:** 10.1192/j.eurpsy.2021.804

**Published:** 2021-08-13

**Authors:** L. Floriano, M. Hainosz, G. Arcaro, C. Brabicoski, E. Krum

**Affiliations:** 1 Nursing And Public Health, State University of Ponta Grossa, Ponta Grossa, Brazil; 2 Pharmacy, State University of Ponta Grossa, Ponta Grossa, Brazil

**Keywords:** mental health, pandemics, Psychological Distress

## Abstract

**Introduction:**

The Coronavirus pandemic has been causing a significant psychological impact on the population, showing symptoms such as anxiety, depression, post-traumatic stress disorder, among others. In addition, health professionals, who are on the front line, need to act promptly seeking unceasingly to save lives, predisposing to psychosocial events due to the risk of contamination, family distance and frustration in relation to death.

**Objectives:**

To analyze the profile of the psychosocial care performed in workers of a Brazilian university hospital who sought care to control the anxiety-stress resulting from the pandemic

**Methods:**

Cross-sectional observational study, using a questionnaire to survey psychosocial demands and evaluate the care of workers who sought assistance in this service (n=61). As a dependent variable, the sector of action was stipulated and as independent the sex, age and the demands that justified the need for assistance. The data were analyzed by Pearson’s correlation with 5% of significance through the statistical software SPSS.

**Results:**

There was a statistic difference between the groups in the variables age and psychosocial interventions for anxiety and stress management. The most part of the sample was composed of health professionals, women, with an average of 33 years old, motivated, technically prepared, scared and not overloaded.

**Conclusions:**

Psychosocial care to health professionals for the management of anxiety and depression is indispensable either during or after the pandemic by seeking Mental Health interventions to minimize the suffering of these workers.

